# Risk management and the perception of the nursing staff on the error in the administration of antimicrobial

**DOI:** 10.1186/1753-6561-5-S6-P156

**Published:** 2011-06-29

**Authors:** M Gaspar, CLD Silva, M Cavalheiro, SN Charneski, OJDC Pipino, S Baglie

**Affiliations:** 1 Departamento de Enfermagem de Saúde Pública, Universidade Estadual de Ponta Grossa - Paraná, Ponta Grossa, Brazil; 2 Santa Casa de Misericórdia de Ponta Grossa, Ponta Grossa, Brazil; 3 Universidade Estadual de Ponta Grossa - Paraná, Ponta Grossa, Brazil; 4 Hospital São Lucas - Pato Branco, Ponta Grossa, Brazil; 5 Departamento de Farmácia, Universidade Estadual de Ponta Grossa - Paraná, Ponta Grossa, Brazil

## Introduction / objectives

The diversity of professionals in the Risk Management has as its aim to expand knowledge and discuss of the facts and risk events and the nurse is an important component of this group, and related to administration of antimicrobial drugs. The objective of this research was to examine the perception of nursing staff in three work shifts, from a health institution in Ponta Grossa – PR, 2009, in relation to the error in the administration of antimicrobials.

## Methods

This is a quantitative research, conducted through a questionnaire with open and closed questions.

## Results

We found that medication error is a multifactor action and the main responsibility is related to the nursing staff. It was found that when antibiotics delays occur in their unit, 14 (58%) do not consider as a medication error, 16 (67%) think it is important to notify the pharmacist and 16 (67%) are aware of the existence of the Nursing and Patient Security Network in Brazil and the Intra-hospital Risk Management Committee and 8 (33%) are unaware about this subject.

## Conclusion

The continuing education for professionals responsible for the administration is the best way of transformation, by encouraging the notification in the register of risk management linked directly with the Committee of Hospital Infection Control of the institution, aiming at quality and safety of care provided to patients.

## Disclosure of interest

None declared.

